# 2,3,4,9-Tetra­hydro-1*H*-carbazole

**DOI:** 10.1107/S1600536808038713

**Published:** 2008-11-26

**Authors:** S. Murugavel, P. S. Kannan, A. SubbiahPandi, T. Surendiran, S. Balasubramanian

**Affiliations:** a Department of Physics, Thanthai Periyar Government Institute of Technology, Vellore 632 002, India; b Department of Physics, SMK Fomra Institute of Technology, Thaiyur, Chennai 603 103, India; c Department of Physics, Presidency College (Autonomous), Chennai 600 005, India; d Department of Chemistry, Sathyabama University, Jeppiaar Nagar, Chennai 600 119, India; e Department of Chemistry, Mohamed Sathak A. J. College of Engineering, Egattur, Chennai 603 103, India

## Abstract

In the title compound, C_12_H_13_N, two methyl­ene C atoms of the cyclo­hexene ring are disordered over two sites with occupancies of 0.591 (10) and 0.409 (10); both disorder components adopt half-chair conformations. The crystal structure is stabilized by inter­molecular N—H⋯π and C—H⋯π inter­actions.

## Related literature

For a related structure, see: Arulmozhi *et al.* (2008 ▶). For general background, see: Mi *et al.* (2003 ▶); Hewlins *et al.* (1984 ▶); Mohanakrishnan & Srinivasan (1995*a* ▶,*b* ▶); Kansal & Potier (1986 ▶); Phillipson & Zenk (1980 ▶); Saxton (1983 ▶); Abraham (1975 ▶). 
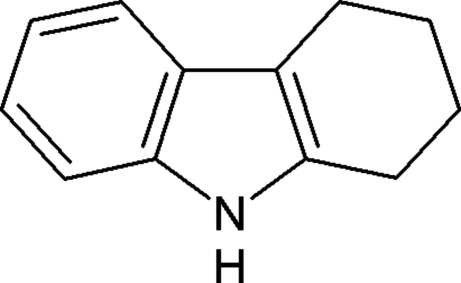


## Experimental

### 

#### Crystal data

C_12_H_13_N
*M* *_r_* = 171.23
Orthorhombic, 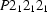

*a* = 6.1067 (4) Å
*b* = 7.9488 (5) Å
*c* = 19.4512 (12) Å
*V* = 944.18 (10) Å^3^
*Z* = 4
Mo *K*α radiation
μ = 0.07 mm^−1^
*T* = 293 (2) K
0.26 × 0.15 × 0.15 mm

#### Data collection

Bruker Kappa APEXII area-detector diffractometer
Absorption correction: none
13269 measured reflections
1777 independent reflections
1323 reflections with *I* > 2σ(*I*)
*R*_int_ = 0.036

#### Refinement

*R*[*F* ^2^ > 2σ(*F* ^2^)] = 0.048
*wR*(*F* ^2^) = 0.123
*S* = 1.07
1777 reflections
137 parameters
15 restraints
H-atom parameters constrained
Δρ_max_ = 0.14 e Å^−3^
Δρ_min_ = −0.20 e Å^−3^

### 

Data collection: *APEX2* (Bruker, 2004 ▶); cell refinement: *SAINT* (Bruker, 2004 ▶); data reduction: *SAINT*; program(s) used to solve structure: *SHELXS97* (Sheldrick, 2008 ▶); program(s) used to refine structure: *SHELXL97* (Sheldrick, 2008 ▶); molecular graphics: *ORTEP-3* (Farrugia, 1997 ▶); software used to prepare material for publication: *SHELXL97* and *PLATON* (Spek, 2003 ▶).

## Supplementary Material

Crystal structure: contains datablocks global, I. DOI: 10.1107/S1600536808038713/ci2708sup1.cif

Structure factors: contains datablocks I. DOI: 10.1107/S1600536808038713/ci2708Isup2.hkl

Additional supplementary materials:  crystallographic information; 3D view; checkCIF report

## Figures and Tables

**Table 1:** Hydrogen-bond geometry (Å, °)

*D*—H⋯*A*	*D*—H	H⋯*A*	*D*⋯*A*	*D*—H⋯*A*
N1—H1*A*⋯*Cg*2^i^	0.86	2.62	3.327 (1)	140
C4—H4⋯*Cg*1^i^	0.93	2.86	3.645 (1)	143
C12—H12*B*⋯*Cg*2^ii^	0.97	2.83	3.577 (2)	135
C12—H12*D*⋯*Cg*2^ii^	0.96	2.72	3.577 (2)	149

Symmetry codes: (i) 
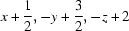
; (ii) 
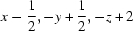
.*Cg*1 and *Cg*2 are the centroids of the N1/C5–C8 and C1–C6 rings, respectively.

**Fig. 1.**
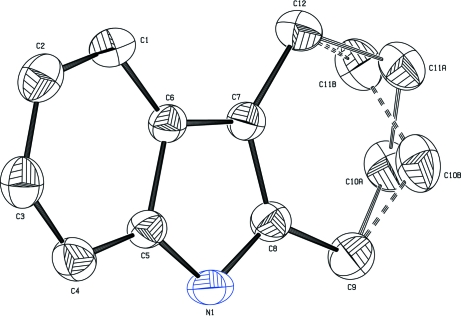
The molecular structure of title compound, showing 30% probability displacement ellipsoids. Both disorder components are shown.

## supplementary crystallographic information

### Comment

Carbazole derivatives exhibit good charge transfer and hole transporting properties, which are being explored for a multitude of optoelectronic and photocatalytic applications, including organic light emitting diodes (OLEDs) (Mi *et al.*, 2003). In carbazole derivatives, the preliminary study shows that the presence of oxygenated substituents increases their biological activity (Hewlins *et al.*, 1984). The 2,3-disubstituted indoles have been used as bidentate synthons for the synthesis of various medicinally important carbazole alkaloids (Mohanakrishnan & Srinivasan, 1995*a*,b). Intercalation between the base pairs in DNA has been implicated for their anticancer activity. It was conceived that the benzo[*b*] carbazoles as isosteric analogs of pyrido[4,3-*b*]carbazoles, with oxygenated D-ring could mimic the anti-cancer activity of ellipticine. So it was of interest to study the anticancer activity of D-ring oxygenated benzo[*b*]carbazoles as it is believed that these molecules could form a stable intercalation complex with DNA (Kansal & Potier, 1986). Tetrahydrocarbazole derivatives are present in the framework of indole-type alkaloids of biological interest (Phillipson & Zenk, 1980; Saxton, 1983; Abraham, 1975). We report here the crystal structure of the title compound (Fig. 1).

Bond lengths are normal and are comparable to the corresponding values observed in 1-naphthyl-9*H*-carbazole-4-sulfonate (Arulmozhi *et al.*, 2008). The dihedral angle between the C1–C6 and N1/C5—C8 rings is 0.6 (1)°. Both the major and minor conformers of the disordered cyclohexene ring adopt half-chair conformations.

The crystal structure is stabilized by intermolecular N—H···π and C–H···π interactions (Table 1).

### Experimental

A mixture of cyclohexanone (0.12 mol) and glacial acetic acid (40 ml) was heated and then redistilled phenylhydrazine (0.1 mol) was added dropwise for 30 min. The mixture was refluxed on a water bath for a further period of 30 min. The reaction mixture was poured into ice-cold water with continuous stirring and brown-coloured solid separated out. It was filtered, washed repeatedly with water and recrystallized from methanol in the presence of a little decolorized carbon to give the title compound. Single crystals suitable for X-ray diffraction were obtained by slow evaporation of a methanol solution.

### Refinement

Atoms C10 and C11 of the cyclohexene ring are disordered over two positions (C10A/C10B and C11A/C11B) with refined occupancies of 0.591 (10) and 0.409 (10). The corresponding bond distances involving the disordered atoms were restrained to be equal. H atoms were positioned geometrically (C—H = 0.93Å and N—H = 0.86%A) and were treated as riding on their parent atoms, with *U*_iso_(H)=1.2*U*_eq_(C,*N*). In the absence of significant anomalous dispersion effects, Friedel pairs were merged before the final refinement.

### Crystal data

**Table:**

C_12_H_13_N	*F*_000_ = 368
*M**_r_* = 171.23	*D*_x_ = 1.205 Mg m^−^^3^
Orthorhombic, *P*2_1_2_1_2_1_	Mo *K*α radiation λ = 0.71073 Å
Hall symbol: P 2ac 2ab	Cell parameters from 1778 reflections
*a* = 6.1067 (4) Å	θ = 2.1–31.1º
*b* = 7.9488 (5) Å	µ = 0.07 mm^−^^1^
*c* = 19.4512 (12) Å	*T* = 293 (2) K
*V* = 944.18 (10) Å^3^	Block, colourless
*Z* = 4	0.26 × 0.15 × 0.15 mm

### Data collection

**Table:**

Bruker Kappa APEXII area-detector diffractometer	1323 reflections with *I* > 2σ(*I*)
Radiation source: fine-focus sealed tube	*R*_int_ = 0.036
Monochromator: graphite	θ_max_ = 31.1º
*T* = 293(2) K	θ_min_ = 2.1º
ω and φ scans	*h* = −8→8
Absorption correction: none	*k* = −11→11
13269 measured reflections	*l* = −28→28
1777 independent reflections	

### Refinement

**Table:**

Refinement on *F*^2^	Secondary atom site location: difference Fourier map
Least-squares matrix: full	Hydrogen site location: inferred from neighbouring sites
*R*[*F*^2^ > 2σ(*F*^2^)] = 0.048	H-atom parameters constrained
*wR*(*F*^2^) = 0.123	*w* = 1/[σ^2^(*F*_o_^2^) + (0.0603*P*)^2^ + 0.0496*P*] where *P* = (*F*_o_^2^ + 2*F*_c_^2^)/3
*S* = 1.07	(Δ/σ)_max_ = 0.001
1777 reflections	Δρ_max_ = 0.14 e Å^−^^3^
137 parameters	Δρ_min_ = −0.20 e Å^−^^3^
15 restraints	Extinction correction: none
Primary atom site location: structure-invariant direct methods	

### Special details

**Table:**

Geometry. All e.s.d.'s (except the e.s.d. in the dihedral angle between two l.s. planes) are estimated using the full covariance matrix. The cell e.s.d.'s are taken into account individually in the estimation of e.s.d.'s in distances, angles and torsion angles; correlations between e.s.d.'s in cell parameters are only used when they are defined by crystal symmetry. An approximate (isotropic) treatment of cell e.s.d.'s is used for estimating e.s.d.'s involving l.s. planes.
Refinement. Refinement of *F*^2^ against ALL reflections. The weighted *R*-factor *wR* and goodness of fit *S* are based on *F*^2^, conventional *R*-factors *R* are based on *F*, with *F* set to zero for negative *F*^2^. The threshold expression of *F*^2^ > σ(*F*^2^) is used only for calculating *R*-factors(gt) *etc*. and is not relevant to the choice of reflections for refinement. *R*-factors based on *F*^2^ are statistically about twice as large as those based on *F*, and *R*- factors based on ALL data will be even larger.

### Fractional atomic coordinates and isotropic or equivalent isotropic displacement parameters (Å^2^)

**Table:**

	*x*	*y*	*z*	*U*_iso_*/*U*_eq_	Occ. (<1)
N1	0.7448 (2)	0.5987 (2)	0.95685 (8)	0.0547 (4)	
H1A	0.8656	0.6513	0.9495	0.066*	
C1	0.3210 (3)	0.4486 (2)	1.06567 (10)	0.0557 (5)	
H1	0.1945	0.3846	1.0604	0.067*	
C2	0.3803 (4)	0.5087 (3)	1.12920 (10)	0.0634 (5)	
H2	0.2921	0.4854	1.1670	0.076*	
C3	0.5689 (4)	0.6033 (3)	1.13826 (10)	0.0619 (5)	
H3	0.6046	0.6419	1.1820	0.074*	
C4	0.7035 (3)	0.6410 (2)	1.08415 (10)	0.0568 (5)	
H4	0.8298	0.7045	1.0904	0.068*	
C5	0.6448 (3)	0.5811 (2)	1.01954 (9)	0.0457 (4)	
C6	0.4535 (3)	0.4849 (2)	1.00903 (9)	0.0429 (4)	
C7	0.4433 (3)	0.4464 (2)	0.93742 (8)	0.0429 (4)	
C8	0.6210 (3)	0.5184 (2)	0.90741 (9)	0.0473 (4)	
C9	0.6730 (3)	0.5153 (3)	0.83301 (10)	0.0668 (6)	
H9A	0.8252	0.4841	0.8267	0.080*	0.591 (10)
H9B	0.6521	0.6267	0.8138	0.080*	0.591 (10)
H9C	0.7893	0.4371	0.8240	0.080*	0.409 (10)
H9D	0.7193	0.6249	0.8182	0.080*	0.409 (10)
C10A	0.5287 (9)	0.3918 (9)	0.7958 (4)	0.0674 (15)	0.591 (10)
H10A	0.5876	0.2795	0.8019	0.101*	0.591 (10)
H10B	0.5318	0.4171	0.7470	0.101*	0.591 (10)
C11A	0.2927 (8)	0.3943 (9)	0.8204 (2)	0.0638 (13)	0.591 (10)
H11A	0.2083	0.3133	0.7941	0.096*	0.591 (10)
H11B	0.2308	0.5049	0.8122	0.096*	0.591 (10)
C10B	0.4709 (17)	0.4587 (13)	0.7953 (5)	0.076 (2)	0.409 (10)
H10C	0.3699	0.5527	0.7924	0.114*	0.409 (10)
H10D	0.5117	0.4282	0.7487	0.114*	0.409 (10)
C11B	0.3543 (15)	0.3125 (11)	0.8276 (3)	0.0651 (19)	0.409 (10)
H11C	0.2313	0.2806	0.7989	0.098*	0.409 (10)
H11D	0.4534	0.2173	0.8300	0.098*	0.409 (10)
C12	0.2741 (3)	0.3519 (3)	0.89757 (10)	0.0579 (5)	
H12A	0.1293	0.3817	0.9141	0.069*	0.591 (10)
H12B	0.2943	0.2320	0.9043	0.069*	0.591 (10)
H12C	0.1428	0.4179	0.8941	0.069*	0.409 (10)
H12D	0.2390	0.2494	0.9212	0.069*	0.409 (10)

### Atomic displacement parameters (Å^2^)

**Table:**

	*U*^11^	*U*^22^	*U*^33^	*U*^12^	*U*^13^	*U*^23^
N1	0.0455 (8)	0.0620 (9)	0.0565 (9)	−0.0161 (8)	0.0044 (7)	0.0019 (7)
C1	0.0518 (10)	0.0556 (11)	0.0597 (11)	−0.0079 (9)	0.0078 (9)	0.0039 (9)
C2	0.0707 (12)	0.0693 (12)	0.0502 (10)	0.0015 (12)	0.0120 (9)	0.0040 (9)
C3	0.0752 (13)	0.0599 (11)	0.0507 (11)	0.0054 (11)	−0.0054 (10)	−0.0048 (9)
C4	0.0577 (11)	0.0495 (10)	0.0631 (12)	−0.0038 (9)	−0.0095 (10)	−0.0022 (9)
C5	0.0447 (8)	0.0402 (8)	0.0522 (9)	−0.0020 (7)	−0.0005 (7)	0.0042 (7)
C6	0.0425 (8)	0.0374 (7)	0.0488 (8)	0.0012 (7)	0.0007 (7)	0.0036 (7)
C7	0.0422 (8)	0.0379 (8)	0.0487 (9)	0.0013 (7)	−0.0007 (7)	0.0010 (7)
C8	0.0440 (8)	0.0468 (9)	0.0511 (9)	0.0018 (8)	0.0023 (7)	0.0017 (8)
C9	0.0601 (11)	0.0876 (15)	0.0526 (10)	−0.0017 (12)	0.0084 (9)	0.0054 (11)
C10A	0.061 (3)	0.082 (4)	0.059 (2)	0.010 (3)	0.004 (2)	−0.007 (3)
C11A	0.057 (2)	0.076 (3)	0.058 (2)	0.003 (2)	−0.0071 (18)	−0.010 (2)
C10B	0.084 (6)	0.097 (6)	0.046 (3)	0.009 (5)	−0.008 (4)	−0.005 (4)
C11B	0.071 (4)	0.067 (4)	0.057 (3)	0.003 (4)	−0.011 (3)	−0.018 (3)
C12	0.0515 (10)	0.0590 (11)	0.0632 (11)	−0.0084 (9)	−0.0038 (9)	−0.0028 (9)

### Geometric parameters (Å, °)

**Table:**

N1—C5	1.371 (2)	C9—H9B	0.97
N1—C8	1.379 (2)	C9—H9C	0.96
N1—H1A	0.86	C9—H9D	0.96
C1—C2	1.374 (3)	C10A—C11A	1.519 (7)
C1—C6	1.397 (2)	C10A—H10A	0.97
C1—H1	0.93	C10A—H10B	0.97
C2—C3	1.387 (3)	C11A—C12	1.542 (5)
C2—H2	0.93	C11A—H11A	0.97
C3—C4	1.369 (3)	C11A—H11B	0.97
C3—H3	0.93	C10B—C11B	1.501 (10)
C4—C5	1.391 (3)	C10B—H10C	0.97
C4—H4	0.93	C10B—H10D	0.97
C5—C6	1.411 (2)	C11B—C12	1.480 (6)
C6—C7	1.427 (2)	C11B—H11C	0.97
C7—C8	1.359 (2)	C11B—H11D	0.97
C7—C12	1.494 (2)	C12—H12A	0.97
C8—C9	1.482 (3)	C12—H12B	0.97
C9—C10B	1.505 (9)	C12—H12C	0.96
C9—C10A	1.505 (6)	C12—H12D	0.96
C9—H9A	0.97		
			
C5—N1—C8	109.19 (14)	H9C—C9—H9D	108.3
C5—N1—H1A	125.4	C9—C10A—C11A	113.3 (5)
C8—N1—H1A	125.4	C9—C10A—H10A	108.9
C2—C1—C6	118.99 (18)	C11A—C10A—H10A	108.9
C2—C1—H1	120.5	C9—C10A—H10B	108.9
C6—C1—H1	120.5	C11A—C10A—H10B	108.9
C1—C2—C3	121.49 (19)	H10A—C10A—H10B	107.7
C1—C2—H2	119.3	C10A—C11A—C12	112.0 (5)
C3—C2—H2	119.3	C10A—C11A—H11A	109.2
C4—C3—C2	121.30 (18)	C12—C11A—H11A	109.2
C4—C3—H3	119.3	C10A—C11A—H11B	109.2
C2—C3—H3	119.3	C12—C11A—H11B	109.2
C3—C4—C5	117.72 (18)	H11A—C11A—H11B	107.9
C3—C4—H4	121.1	C11B—C10B—C9	114.6 (7)
C5—C4—H4	121.1	C11B—C10B—H10C	108.6
N1—C5—C4	130.84 (17)	C9—C10B—H10C	108.6
N1—C5—C6	107.17 (15)	C11B—C10B—H10D	108.6
C4—C5—C6	121.99 (17)	C9—C10B—H10D	108.6
C1—C6—C5	118.50 (16)	H10C—C10B—H10D	107.6
C1—C6—C7	134.42 (16)	C12—C11B—C10B	112.2 (7)
C5—C6—C7	107.08 (15)	C12—C11B—H11C	109.2
C8—C7—C6	107.10 (14)	C10B—C11B—H11C	109.2
C8—C7—C12	122.77 (16)	C12—C11B—H11D	109.2
C6—C7—C12	130.10 (15)	C10B—C11B—H11D	109.2
C7—C8—N1	109.45 (15)	H11C—C11B—H11D	107.9
C7—C8—C9	125.70 (17)	C11B—C12—C7	110.8 (3)
N1—C8—C9	124.85 (17)	C7—C12—C11A	110.1 (2)
C8—C9—C10B	107.8 (4)	C11B—C12—H12A	131.1
C8—C9—C10A	110.8 (3)	C7—C12—H12A	109.6
C8—C9—H9A	109.5	C11A—C12—H12A	109.6
C10B—C9—H9A	130.4	C11B—C12—H12B	82.7
C10A—C9—H9A	109.5	C7—C12—H12B	109.6
C8—C9—H9B	109.5	C11A—C12—H12B	109.6
C10B—C9—H9B	88.7	H12A—C12—H12B	108.1
C10A—C9—H9B	109.5	C11B—C12—H12C	109.1
H9A—C9—H9B	108.1	C7—C12—H12C	109.8
C8—C9—H9C	110.3	C11A—C12—H12C	82.8
C10B—C9—H9C	109.0	H12B—C12—H12C	130.7
C10A—C9—H9C	85.6	C11B—C12—H12D	109.5
H9B—C9—H9C	128.2	C7—C12—H12D	109.4
C8—C9—H9D	109.9	C11A—C12—H12D	131.9
C10B—C9—H9D	111.5	H12A—C12—H12D	81.1
C10A—C9—H9D	128.3	H12C—C12—H12D	108.1
H9A—C9—H9D	84.9		
			
C6—C1—C2—C3	−0.4 (3)	C5—N1—C8—C7	−1.0 (2)
C1—C2—C3—C4	0.1 (3)	C5—N1—C8—C9	177.78 (18)
C2—C3—C4—C5	0.0 (3)	C7—C8—C9—C10B	14.1 (5)
C8—N1—C5—C4	−178.92 (19)	N1—C8—C9—C10B	−164.5 (5)
C8—N1—C5—C6	0.7 (2)	C7—C8—C9—C10A	−11.7 (4)
C3—C4—C5—N1	179.77 (19)	N1—C8—C9—C10A	169.7 (3)
C3—C4—C5—C6	0.2 (3)	C8—C9—C10A—C11A	40.6 (8)
C2—C1—C6—C5	0.6 (3)	C10B—C9—C10A—C11A	−46.8 (11)
C2—C1—C6—C7	−179.34 (19)	C9—C10A—C11A—C12	−59.6 (9)
N1—C5—C6—C1	179.87 (15)	C8—C9—C10B—C11B	−43.8 (11)
C4—C5—C6—C1	−0.4 (3)	C10A—C9—C10B—C11B	57.5 (12)
N1—C5—C6—C7	−0.20 (18)	C9—C10B—C11B—C12	61.8 (14)
C4—C5—C6—C7	179.48 (17)	C10B—C11B—C12—C7	−42.9 (10)
C1—C6—C7—C8	179.52 (19)	C10B—C11B—C12—C11A	51.4 (8)
C5—C6—C7—C8	−0.39 (18)	C8—C7—C12—C11B	14.2 (5)
C1—C6—C7—C12	1.6 (3)	C6—C7—C12—C11B	−168.1 (5)
C5—C6—C7—C12	−178.35 (17)	C8—C7—C12—C11A	−17.0 (4)
C6—C7—C8—N1	0.84 (19)	C6—C7—C12—C11A	160.7 (3)
C12—C7—C8—N1	178.98 (16)	C10A—C11A—C12—C11B	−51.7 (7)
C6—C7—C8—C9	−177.92 (18)	C10A—C11A—C12—C7	45.1 (7)
C12—C7—C8—C9	0.2 (3)		

### Hydrogen-bond geometry (Å, °)

**Table:**

*D*—H···*A*	*D*—H	H···*A*	*D*···*A*	*D*—H···*A*
N1—H1A···Cg2^i^	0.86	2.62	3.327 (1)	140
C4—H4···Cg1^i^	0.93	2.86	3.645 (1)	143
C12—H12B···Cg2^ii^	0.97	2.83	3.577 (2)	135
C12—H12D···Cg2^ii^	0.96	2.72	3.577 (2)	149

Symmetry codes: (i) *x*+1/2, −*y*+3/2, −*z*+2; (ii) *x*−1/2, −*y*+1/2, −*z*+2.
